# Atypical presentations in the hospitalised older adult testing positive for SARS-CoV-2: a retrospective observational study in Glasgow, Scotland

**DOI:** 10.1177/0036933020962891

**Published:** 2020-10-11

**Authors:** Peter Davis, Rory Gibson, Emily Wright, Amy Bryan, Jamie Ingram, Ren Ping Lee, Jon Godwin, Tom Evans, Elizabeth Burleigh, Steven Wishart, Eileen Capek, Lara Mitchell

**Affiliations:** 1 Doctor, Department of Medicine for the Elderly, Queen Elizabeth University Hospital, UK; 2 Professor of Statistics, Glasgow Caledonian University, UK; 3 Professor of Molecular Microbiology, University of Glasgow, UK; 4 Consultant Physician, Department of Medicine for the Elderly, Queen Elizabeth University Hospital, UK

**Keywords:** Severe Acute Respiratory Syndrome Coronavirus 2, Coronavirus Disease 2019, delirium, prognostic indicators, older people

## Abstract

**Introduction:** Understanding of how SARS-CoV-2 manifests itself in older adults was unknown at the outset of the pandemic. We undertook a retrospective observational analysis of all patients admitted to older people’s services with confirmed COVID-19 in one of the largest hospitals in Europe. We detail presenting symptoms, prognostic features and vulnerability to nosocomial spread. **Methods:** We retrospectively collected data for each patient with a positive SARSCoV-2 RT PCR between 18th March and the 20th April 2020 in a department of medicine for the elderly in Glasgow. **Results:** 222 patients were included in our analysis. Age ranged from 56 to 99 years (mean = 82) and 148 were female (67%). 119 patients had a positive swab for SARS-CoV-2 within the first 14 days of admission, only 32% of these patients presented with primarily a respiratory type illness. 103 patients (46%) tested positive after 14 days of admission – this was felt to represent likely nosocomial infection. 95 patients (43%) died by day 30 after diagnosis. **Discussion:** This data indicates that older people were more likely to present with non-respiratory symptoms. High clinical frailty scores, severe lymphopenia and cumulative comorbidities were associated with higher mortality rates. Several contributing factors will have led to nosocomial transmission.

## Introduction

Since first emerging in Wuhan in 2019, the novel coronavirus SARS-CoV-2, which causes COVID-19 disease, has reached global pandemic proportions. The rapid spread, novel features and global differences in public health approaches have led to worldwide challenges in caring for patients as individuals whilst formulating population-wide strategies to address the crisis. We present data on a large group of hospitalised older adults during the COVID-19 outbreak in Scotland. The majority were aged over 65, with a subset of younger people who were undergoing specialist stroke rehabilitation. Our data includes the peaks for number of inpatients in Scotland with COVID-19 (31st March) and highest mortality rate for COVID-19 in Scotland (9th April).^1^

The initial Health Protection Scotland (HPS) case definition of COVID-19 requiring hospital admission included clinical or radiological evidence of pneumonia, respiratory distress or a fever of ≥37.8°C with a new cough or other upper respiratory tract symptom. Anosmia and/or altered taste was added later.^2^ This definition is important as it guides clinicians on who to test for SARS-CoV-2. Initial reports from Wuhan had suggested over 90% of patients presented with a cough^3^ and >60% had fever.^4^ However, recognition of how COVID-19 presents in the older adult has evolved during this pandemic and it is increasingly recognised that older adults are more likely to have an atypical presentation. Recent case reports have highlighted presentations including falls or delirium without fever^5^ and gastrointestinal symptoms. Diagnostic uncertainty causes an increased risk of missed diagnosis if clinicians do not have a high index of suspicion and awareness of these atypical presentations.

Patients over the age of 70 are more likely to have multiple co-morbidities and are shown to have a higher rate of mortality than younger patients when testing positive for SARS-CoV-2.^6^ Current data suggests a PCR sensitivity of 70–90%^7^ giving a significant number of false negative results. It is therefore critical that we identify COVID-19 in elderly patients admitted to hospital in order to manage them effectively and minimise risk of transmission to staff and patients.

The aims of this retrospective analysis were to identify presenting symptoms, comorbidities, clinical frailty scores (CFS), biomarkers and radiological findings in the older adult with SARS-CoV-2 infection. Furthermore, we aimed to identify associations between these factors and patient outcomes.

## Methodology

Our team conducted a retrospective observational analysis of all patients with laboratory confirmed SARS-CoV-2 admitted to older people services in south sector of NHS Greater Glasgow and Clyde between 18th March and 20th April 2020 inclusive. This comprises 17 wards across three sites in Glasgow; the Queen Elizabeth University Hospital (QEUH), Gartnavel General Hospital (GGH) and the New Victoria Hospital (NVH). In total there are 456 inpatient beds across these sites including acute receiving areas (16 beds), acute assessment elderly care (196 beds), stroke and rehabilitation wards. Two thirds of patients are in open, 3–6 bedded bays alongside a total of 150 side rooms. Patients are initially admitted through the Emergency Department or GP receiving unit at the QEUH. If required, patients subsequently move to offsite rehabilitation wards at GGH and NVH.

## Data collection

Approval from the local Cauldicott guardian was obtained prior to commencing this study. We reviewed inpatient notes, laboratory results, radiology reports, allied health practitioner notes and previous hospital records including GP referrals. We used this information to collect data on existing comorbidities, 4AT score, baseline CFS, investigations at point of first positive swab, documented suspicion of COVID-19 and the clinical outcome including 30-day mortality from swab date. Symptoms which developed after the swab were not. 6 doctors performed data collection in a standardised electronic data collection form. Discrepancies in data collection were minimised by a prior calibrating session to ensure information was interpreted uniformly by each collector. Primary outcome measures recorded were death, length of stay in hospital and discharge destination.

## Laboratory procedures

WHO guidelines states laboratory confirmation for SARS-CoV-2 is from a RT PCR of a swab taken from the nasopharynx.^8^ Samples were analysed at the West of Scotland Specialist Virology Centre located at the Glasgow Royal Infirmary. At the time data was collected, patients were swabbed and isolated based on clinical suspicion.

## Definitions

Fever was defined as a temperature of ≥37.8°C. AKI was defined by a serum creatinine rise of at least 50% from baseline as per NICE.^9^ The British Society of Thoracic Imaging (BSTI) have recommended the CVCX scoring system for standardised reporting of chest x-rays (CXR) during this pandemic, which was routinely used CXRs reported after March 2020.^10^ We have also included these scores in our data set. Cardiovascular disease was defined as a history of ischaemic heart disease, peripheral vascular disease or stroke. Gastrointestinal upset included nausea, diarrhoea, anorexia or abdominal pain. Shortness of breath or raised respiratory rate was felt to represent respiratory distress.

## Statistical analysis

Fishers exact test was used for all dichotomous variables, either Chi-Square for trend or the Mann-Whitney U test were used for ordinal series. All p values are “two-tailed”.

## Results

A total of 232 patients were identified for possible inclusion into our analysis. 3 were excluded due to their clinical notes being unavailable at time of data collection. A further 7 were excluded because they first tested positive after being discharged from our department. 5 patients were tested in the community prior to admission; these patients have been included. A total of 222 patients were included into our final analysis. Ages ranged from 56 to 99 years (mean = 82) and 148 (67%) were female.

For the purposes of analysis, the cohort was divided into 2 subgroups; patients that tested positive ≤14 days of admission (n = 119, Group 1) and patients that first tested positive after 14 days of admission which most likely represented nosocomial cases (n = 103, Group 2). Table 1 summarises patient demographics, clinical characteristics, investigations and outcomes.

**Table 1.** Summary of baseline clinical characteristics, symptoms, biochemical markers and outcome.

Patient Demographics and pre-existing characteristics	All patients (n = 222)	Group 1Patients swabbed ≤14 days of admission (n = 119)	Group 2Patients swabbed >14 days of admission (n = 103)	p-Value
Female	148 (67%)	83 (70%)	65 (63%)	
Male	74 (33%)	36 (30%)	38 (37%)	
Mean Age	82	82	82	
Age range	56–99	56–96	58–99	
Place of admission				
Nursing Home	27 (12%)	23 (19%)	4 (4%)	0.0003
Residential Home	7 (3%)	5 (4%)	2 (2%)	0.3
Sheltered accommodation	8 (4%)	3 (3%)	5 (5%)	0.5
Admitted from ‘other'	2 (1%)	2 (2%)	0 (0%)	0.2
Own home	178 (80%)	86 (72%)	92 (89%)	0.7
Comorbidities				
Dementia	86 (39%)	49 (41%)	37 (36%)	0.2
Cardiovascular disease	113 (51%)	61 (51%)	52 (50%)	0.4
Hypertension	125 (56%)	63 (53%)	62 (60%)	0.9
Diabetes	48 (22%)	28 (24%)	20 (19%)	0.2
COPD/Asthma	48 (22%)	25 (21%)	23 (22%)	0.8
Clinical Frailty Score				
Mean CFS	5.22	5.30	5.10	
CFS range	1 to 8	2 to 8	1 to 7	
1	1 (0%)	0 (0%)	1 (1%)	
2	7 (3%)	3 (3%)	4 (4%)	
3	16 (7%)	9 (8%)	7 (7%)	
4	31 (14%)	15 (13%)	16 (16%)	
5	57 (26%)	31 (26%)	26 (25%)	
6	72 (32%)	42 (35%)	30 (29%)	
7	37 (17%)	18 (15%)	19 (18%)	
8	1 (0%)	1 (1%)	0 (0%)	
Presenting characteristics Core Symptoms				
Fever (≥37.8)	82 (37%)	46 (39%)	36 (35%)	0.3
Cough	118 (53%)	68 (57%)	50 (49%)	0.1
Respiratory distress	64 (29%)	52 (44%)	12 (12%)	<0.00001
GI upset	36 (16%)	23 (19%)	13 (13%)	0.1
Myalgia	13 (6%)	9 (8%)	4 (4%)	0.2
No recorded symptoms	17 (8%)	6 (5%)	11 (11%)	0.2
Predominant other symptom				
Chest pain	6 (3%)	5 (4%)	1 (1%)	0.1
Delirium	70 (32%)	39 (33%)	31 (30%)	0.3
Falls	13 (6%)	12 (10%)	1 (1%)	0.002
Malaise	20 (9%)	16 (13%)	4 (4%)	0.007
Coryzal symptoms	10 (5%)	8 (7%)	2 (2%)	0.06
Presyncope/syncope	3 (1%)	2 (2%)	1 (1%)	0.6
Other	17 (8%)	11 (9%)	6 (6%)	0.2
4AT				
Not recorded	39 (18%)	4 (3%)	35 (34%)	
0	48 (22%)	29 (24%)	19 (18%)	
1 to 3	36 (16%)	28 (24%)	8 (8%)	
≥4	99 (45%)	58 (49%)	41 (40%)	
Laboratory and radiological findings				
Lymphocytes (×10^9^/L)				
Not recorded	23 (10%)	1 (1%)	22 (21%)	
<0.5	22 (10%)	15 (13%)	7 (7%)	
0.5–1	98 (44%)	57 (48%)	41 (40%)	
1.1–1.5	46 (21%)	24 (20%)	22 (21%)	
>1.5	33 (15%)	22 (18%)	11 (11%)	
Abnormal AST/ALT				
Not recorded	25 (11%)	1 (1%)	24 (23%)	
Yes	48 (22%)	38 (32%)	10 (10%)	
No	149 (67%)	80 (67%)	69 (67%)	
CRP (mg/L)				
Not recorded	20 (9%)	1 (1%)	19 (18%)	
0–10	30 (14%)	15 (13%)	15 (15%)	
11 to 50	75 (34%)	35 (29%)	40 (39%)	
51–100	48 (22%)	27 (23%)	21 (20%)	
101–200	34 (15%)	28 (24%)	6 (6%)	
201–300	10 (5%)	9 (8%)	1 (1%)	
300+	5 (2%)	4 (3%)	1 (1%)	
Na (mmol/L)				
Not recorded	20 (9%)	1 (1%)	19 (18%)	
<125	0 (0%)	0 (0%)	0 (0%)	
125–129	13 (6%)	6 (5%)	7 (7%)	
130–135	33 (15%)	19 (16%)	14 (14%)	
>135	156 (70%)	93 (78%)	63 (61%)	
AKI				
Not recorded	20 (9%)	1 (1%)	19 (18%)	
Yes	45 (20%)	28 (24%)	17 (17%)	
No	157 (71%)	90 (76%)	67 (65%)	
CVCX				
Not recorded	65 (29%)	12 (10%)	53 (51%)	
0	67 (30%)	42 (35%)	25 (24%)	
1	32 (14%)	27 (23%)	5 (5%)	
2	58 (26%)	38 (32%)	20 (19%)	
Outcome after 30 days				
Treated in critical care at any point of admission	4 (2%)	0 (0%)	4 (4%)	0.05
Death	95 (43%)	54 (45%)	41 (40%)	0.2
Discharged to current residence	63 (28%)	49 (41%)	18 (17%)	0.0002
Discharged to a new residence	11 (5%)	2 (2%)	9 (9%)	0.03
Remained IP	49 (22%)	14 (12%)	35 (34%)	0.003
Average length of stay (excludes current IP)	35.35	12.12	70.88	<0.001

173 (78%) patients had one or more of the following symptoms: fever, cough or respiratory distress. 17 (8%) patients were reported to have no documented symptoms. Delirium was diagnosed in 70 (32%) patients.

**Figure 1.**
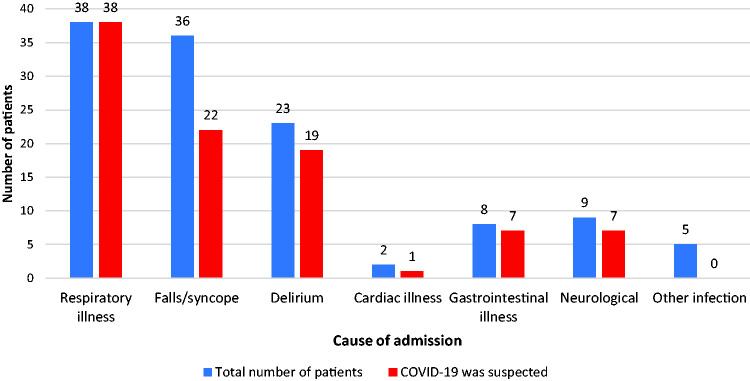
Presentation of group 1 patients and whether COVID-19 was suspected within first 24 hours of admission.

Such atypical presentations or lack of symptoms led to diagnostic uncertainty. Figure 1 shows the primary reason for hospitalisation of Group 1 patients and whether COVID-19 was listed as a possible differential diagnosis by the admitting doctor. COVID-19 was not suspected in 28 (19%) patients within this group.

 Table 2 describes various clinical characteristics, biomarkers and radiological findings and their association with 30-day mortality. Profound lymphopenia, high CRP, high clinical frailty score and cumulative morbidity were associated with a higher chance of mortality.

**Table 2.** Clinical characteristics and associated mortality.

	Number of patients	Deaths	Mortality rate	2p-Value
Gender				
Female	148	61	41.22%	
Male	74	34	45.95%	
Clinical frailty score				
CFS ≤4	55	18	32.73%	
CFS ≥5	167	77	46.11%	
1	1	0	0.00%	
2	7	1	14.29%	
3	16	4	25.00%	
4	31	13	41.94%	
5	57	21	36.84%	
6	72	32	44.44%	
7	37	23	62.16%	
8	1	1	100.00%	
Place of residence				
NH resident	27	21	77.78%	0.004
Residential Home	7	3	42.86%	0.7
Sheltered accommodation	8	2	25.00%	0.2
Admitted from ‘other'	2	1	50.00%	1
Own home	178	53	29.78%	<0.00001
Comorbidity				
Dementia	86	43	50.00%	0.03
Cardiovascular disease	113	54	47.79%	0.5
Hypertension	125	56	44.80%	0.4
Diabetes	48	26	54.17%	0.2
COPD/Asthma	48	19	39.58%	0.7
CVCX				
Not recorded	65			
0	67	22	32.84%	
1	32	23	71.88%	
2	58	30	51.72%	
Lymphocytes (×10^9^/L)				
Not recorded	23			
<0.5	22	14	63.64%	
0.5–1	98	45	45.92%	
1.1–1.5	46	16	34.78%	
>1.5	33	13	39.39%	
Abnormal AST/ALT (U/L)				
Not recorded	25			
Yes	48	24	50.00%	
No	149	64	42.95%	
C-reactive protein (mg/L)				
Not recorded	20			
0–10	30	6	20%	
11 to 50	75	22	29%	
51–100	48	29	60%	
101–200	34	24	71%	
201–300	10	4	40%	
300+	5	5	100%	
Serum sodium (mmol/L)				
Not recorded	20			
<125	0			
125–129	13	2	15.38%	
130–135	33	8	24.24%	
>135	156	80	51.28%	
Acute kidney injury				
Not recorded	20			
Yes	45	28	62.22%	
No	157	62	39.49%	
Number of comorbidities				
0	12	3	25.00%	
1	80	33	41.25%	
2	67	24	35.82%	
3	47	24	51.06%	
4	15	10	66.67%	
4AT				
Not recorded	39			
0	48	16	33.33%	
1 to 3	36	11	30.56%	
≥4	99	51	51.52%	

**Figure 2.**
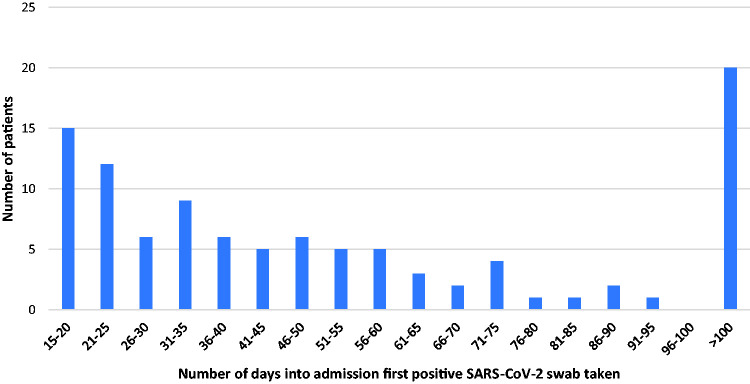
Length of hospital admission of Group 2 patients prior to first positive swab.

 Figure 2 demonstrates the length of admission prior to first positive swab for group 2 patients. Day 0 was taken as day of admission. 97 (82%) of Group 1 patients had their first positive swab with 24 hours of admission.

## Discussion

Older patients are just as likely to present to hospital with SARS-CoV-2 with falls/syncope or delirium as they would with primarily a respiratory type illness. Clinicians were good at recognising COVID in respiratory illness; all patients who presented with a respiratory type illness in Group 1 had COVID-19 documented as a differential diagnosis. However, it was only suspected in 61% of cases if they presented with a fall and/or syncope. 26 (12%) patients at time of positive swab had no fever or respiratory distress and a CVCX score of 0 – thus not fulfilling the HPS case definition at that time. 17 patients (8%) had no documented symptoms whatsoever. These patients may have been swabbed at a ‘sub-clinical’ phase of infection or have had asymptomatic carriage.^11^ We suspect if we had performed surveillance swabs at this time, the proportion of asymptomatic/atypical symptoms would be higher.

Overall, our study highlights the difficulties in identifying SARS-CoV-2 in this patient group; it is extremely difficult to exclude COVID-19 on the basis of clinical features only. Almost a fifth of patients within the cohort were not initially suspected of having SARS-CoV-2 infection. In the earlier phase of the pandemic there was poorer understanding of the clinical spectrum of symptoms and atypical presentation of the disease; this has evolved over time. Furthermore, differential diagnosis was influenced, and continues to be, by the HPS case definition which arguably does not adequately reflect SARS-CoV-2 infection in the older adult. SIGN guidelines have recently been updated to reflect this. However, public health definitions have not changed; this needs to be debated nationally as a priority.

Fever (at point of swab) was found in 37% which is less common than in previous studies. This can be partly explained by our definition of fever, as previous studies in China have used a temperature of >37.3,^12^ other European studies have reported a lower proportion of 45% presenting with fever.^13^ If the patient developed fever later in their admission, this was not included in our data. A lack of fever could potentially be as a result of blunting of typical inflammatory febrile response due to changes in thermoregulation and immune cell dysregulation in the older adult which has been postulated in other literature. It is also widely observed that a febrile response does not occur in acute infection in around 20% of older people.^14^

Delirium was diagnosed in a third of our total patient group. Of the 183 with a 4AT score documented, 99 (54%) had a score of ≥4. A 4AT score of ≥4 was associated with a higher 30-day mortality (2p = 0.0002). It is reasonable to assume that those with a 4AT score documented were more likely to have a suspected delirium than those without one documented. 4AT is an important tool in diagnosing delirium - existing data suggests without screening for delirium roughly 75% of cases can be missed in inpatients.^15^ A recent meta-analysis found a total occurrence of delirium in the hospitalised adult (average age range of studies 66-87) to be around 23% on medical wards.^16^ There is limited data describing delirium associated with SARS-CoV-2. A case series in London suggested 24 of 101 patients referred to palliative care had symptoms suggestive of delirium.^17^ Dementia was a common comorbidity in our patient group; 86 patients had a pre-existing diagnosis of dementia. Of the 70 patients diagnosed with delirium, 25 had a pre-existing diagnosis of dementia, as did 53 of the 99 patients with a 4AT of ≥4. Dementia was associated with a higher mortality rate (2p = 0.03).

NICE have recommended using clinical frailty scale in patients aged 65 or older with regards to decisions about admission to intensive care units^18^ and have produced guidelines suggesting that patients with COVID-19 and a CFS ≥5 would not benefit from ITU admission. Patients aged >80 with a CFS score ≥5 admitted with non COVID-19 related conditions to ITU in Australia had a significantly poorer health outcome.^19^ Our data shows a clinical frailty score of 5 or above was associated with a higher chance of mortality (2p = 0.02), which likely reflects a lack of physiological reserve in these older and comorbid patients. This may aid future decision making regarding critical care admissions for patients in our age group.

Age is a predictor of mortality from COVID-19 which been consistently shown to be >20% for the over 80 years old.^20^ Interestingly, within our cohort of patients, age was not a predictor of mortality. We would support an individualised assessment to make decisions requiring critical care admission including comorbidities and clinical frailty scores rather than an age-based approach.

Lymphopenia (≤1 × 10^9^/l) occurred in 60% of patients in our data, which is similar to previous studies in China.^21^ A severe lymphopenia (≤0.5 × 10^9^/l) was associated with a higher mortality rate (2p = 0.02). This supports previous studies in Wuhan^22^ suggesting lymphopenia as a prognostic indicator. Patients with ‘classical’ chest x-ray changes (CVCX1) had a mortality rate of 72% when compared with a ‘normal’ chest x-ray (CVCX0) mortality rate of 33% (2p = 0.0006). A bilateral pneumonia is likely a reflection of severity of disease.

Patients from nursing and residential homes represented 15% of our patient group. The mortality rate for nursing home patients was particularly high (78%), which could be explained by a high threshold of admission from a nursing home and their pre-existing frailty. This is additionally important for informing community anticipatory care planning in the event of further spikes.

46% of our cases were patients who had their first positive swab following more than 14 days of admission. This could be interpreted as nosocomial cases, although this definition is yet to be formally established. Factors contributing include but are not limited to: delay in diagnosis due to atypical presentation, evolving personal protective equipment use in the early stages of pandemic, patient movement within wards and the wider hospital, availability of single rooms, challenges in containing the ‘wandering patient’, visitors to hospitals (ceased on the 28th March 2020) and likely asymptomatic spread. Blanket testing for all newly admitted patients aged 70 and over (and every 4 days thereafter) was only introduced on 29th April 2020. Regular and widespread testing (of both patients and staff) along with robust infection control protocols will be key in the event of further outbreaks within hospital settings. We would suggest that similarities can be found in the Scottish care home populations, where over 1,800 deaths have been recorded as COVID-19 related as of the first week of June 2020.^23^ Both populations arguably represent the top quartile of frailty with a high burden of comorbidities, cognitive and function impairment.

## Study limitations

We recognise there are limitations of our study. Several patients were tested without clear clinical documentation as to why the swab was done; it is unclear whether these patients were intentionally swabbed or had undocumented symptoms. A high proportion of patients presented with delirium and 39% had dementia: self-reported symptoms may therefore be unreliable. Our patient cohort represents some of the frailest members of society, at least 95% of whom had a significant co-morbidity. It is therefore not representative of all older people and must be contextualised as such.

## Conclusion

This study demonstrates that the older adult with SARS-CoV-2 infection is more likely to present to hospital with atypical symptomology. Falls and delirium commonly precipitate admission as well as classical respiratory symptoms. Almost a fifth of the cohort were not initially suspected as having SARS-CoV-2 infection. Although SIGN guidelines have recently been updated to recognise the atypical presentation in the older adult, public health definitions have not changed; this needs to be debated nationally as a priority. A clinical frailty score ≥5, severe lymphopenia, cumulative comorbidities and high CRP was associated with a higher mortality rate. Age was not a predictor of death in this cohort. Nosocomial infection accounted for almost half of our cohort – future priorities should focus on robust testing protocols within hospitals including point of care testing.
